# Combined Transradial and Transfemoral Approach With Ostial Vertebral Balloon Protection for the Treatment of Patients With Subclavian Steal Syndrome

**DOI:** 10.3389/fneur.2020.576383

**Published:** 2020-10-22

**Authors:** Rami Fakih, Sudeepta Dandapat, Alan Mendez-Ruiz, Aldo A. Mendez, Mudassir Farooqui, Cynthia Zevallos, Darko Quispe Orozco, David Hasan, James Rossen, Edgar A. Samaniego, Colin Derdeyn, Santiago Ortega-Gutierrez

**Affiliations:** ^1^Department of Neurology, University of Iowa Hospitals and Clinics, Iowa City, IA, United States; ^2^Department of Neurosurgery, University of Iowa Hospitals and Clinics, Iowa City, IA, United States; ^3^Department of Internal Medicine—Cardiovascular Medicine, University of Iowa Hospitals and Clinics, Iowa City, IA, United States; ^4^Department of Radiology, University of Iowa Hospitals and Clinics, Iowa City, IA, United States

**Keywords:** subclavian steal syndrome, distal embolic protection, SAPTA, subclavian artery occlusion, subclavian angioplasty

## Abstract

**Background:** Patients with an obstructive subclavian artery (SA) may exhibit symptoms of vertebrobasilar insufficiency known as subclavian steal syndrome (SSS). Endovascular treatment with stent assisted percutaneous transluminal angioplasty (SAPTA) demonstrates significantly lower percentage of intraoperative and postoperative complications in comparison with open surgery. There is a 1–5% risk of distal intracranial embolization through the ipsilateral vertebral artery (VA) during SAPTA.

**Objective:** To assess the safety and feasibility of a novel technique for distal embolic protection using balloon catheters during SA revascularization with a dual transfemoral and transradial access.

**Methods:** We describe a case series of patients with SSS who underwent SAPTA due to severe stenosis or occlusion of the SA using a combined anterograde/retrograde approach. Transfemoral access to SA was obtained using large bore guide sheaths. Ipsilateral transradial access was obtained using intermediate bore catheters. A Scepter XC balloon catheter was introduced through the transradial intermediate catheter into the ipsilateral VA at the ostium during SAPTA for distal embolic protection.

**Results:** A total of eight patients with SSS underwent subclavian SAPTA. Four patients had the combined anterograde/retrograde approach. Successful revascularization was achieved in three of them. It was difficult to create a channel in the fourth unsuccessful case due to heavily calcified plaque burden. No peri-operative ischemic events were identified. On follow-up, we demonstrated patency of the stents with resolution of symptoms and without any adverse events.

**Conclusion:** Subclavian stenting using a combined transradial and transfemoral access with compliant balloon catheters at the vertebral ostium for prevention of distal emboli may represent an alternative therapeutic approach for the treatment of SA stenosis and occlusions.

## Introduction

Subclavian steal syndrome (SSS) is caused by a reversal of flow in the vertebral artery (VA) ipsilateral to a stenosis or occlusion of the prevertebral subclavian artery (SA) (1). Patients with obstructive SA may exhibit upper extremity claudication, syncope, dizziness, or arm coolness owing to arterial insufficiency in the brain (vertebrobasilar insufficiency) or upper extremity, which are both supplied by the SA (1–3). The most common cause of SA stenosis is atherosclerosis (3).

The standard practice consists in conservative management for asymptomatic patients while interventions are performed for patients suffering from SSS (3, 4). Treatment of SA steno-occlusive disease includes either extra-thoracic surgical approaches, or endovascular approaches through stent assisted percutaneous transluminal angioplasty (SAPTA). Extra-thoracic operations (subclavian-carotid transposition and carotid-subclavian bypass) for the treatment of SA stenosis and occlusions have been historically performed (3–5). However, recently endovascular therapy has been the first line treatment of SA stenosis (4–7). In comparison with open surgery, endovascular technology demonstrates significantly lower percentage of intraoperative and postoperative complications, and it is carried out under local anesthesia (4, 8, 9). Nowadays, an endovascular approach is attempted first before proceeding to open SA revascularization as it is a less invasive procedure (5, 9).

Most of neuroendovascular interventions are performed *via* a transfemoral approach. Transradial access is an alternative approach for neuroendovascular procedures that lately is being more employed (10, 11). Advantages of radial access include decreased cost and patient preference for post-operative recovery (12). For SA steno-occlusive disease, it provides added advantage with regards to catheterizing the true vessel lumen (6, 10, 13). There is 1–5% risk of stroke during SAPTA of SA (14–16). Distal embolization through the ipsilateral VA to the posterior circulation is one of the major concerns, which can lead to periprocedural stroke. We report four consecutive cases of symptomatic SA occlusion/near occlusions with varying degrees of stenosis, which were treated at our center using a novel combined endovascular technique with both anterograde (transfemoral) and retrograde (transradial) access to SA, while using a balloon catheter in the ipsilateral VA ostium for distal emboli protection.

## Methods

### Study Population

We performed a retrospective search of our prospectively acquired endovascular intervention database. All cases of SA stenosis treated with endovascular intervention were assessed for inclusion and their medical charts were reviewed. Inclusion criteria were patients with diagnosis of SSS who underwent endovascular treatment for occlusion of the SA at our center from 2014 to June 2019. Symptoms of SSS were defined as vertebrobasilar insufficiency symptoms such as dizziness, syncope, nausea, dysmetria, as well as upper extremity claudication/weakness. Subclavian steal phenomena on imaging was defined as critical stenosis or occlusion of the SA with retrograde flow into the ipsilateral VA from the contralateral VA as seen on digital subtraction angiography (DSA). Patients were included in the study only if they had symptoms of vertebrobasilar insufficiency that was explained by subclavian steal phenomena seen on imaging. Catheter and stent selection were decided by the institutional neuro-interventionalist. *Ethics:* Written informed consent was obtained from the individual/next of kin for the publication of any potentially identifiable images or data included in this article. Approval for the study was obtained from our institutional review board (IRB).

### Interventional Procedure

**Figure 1:**
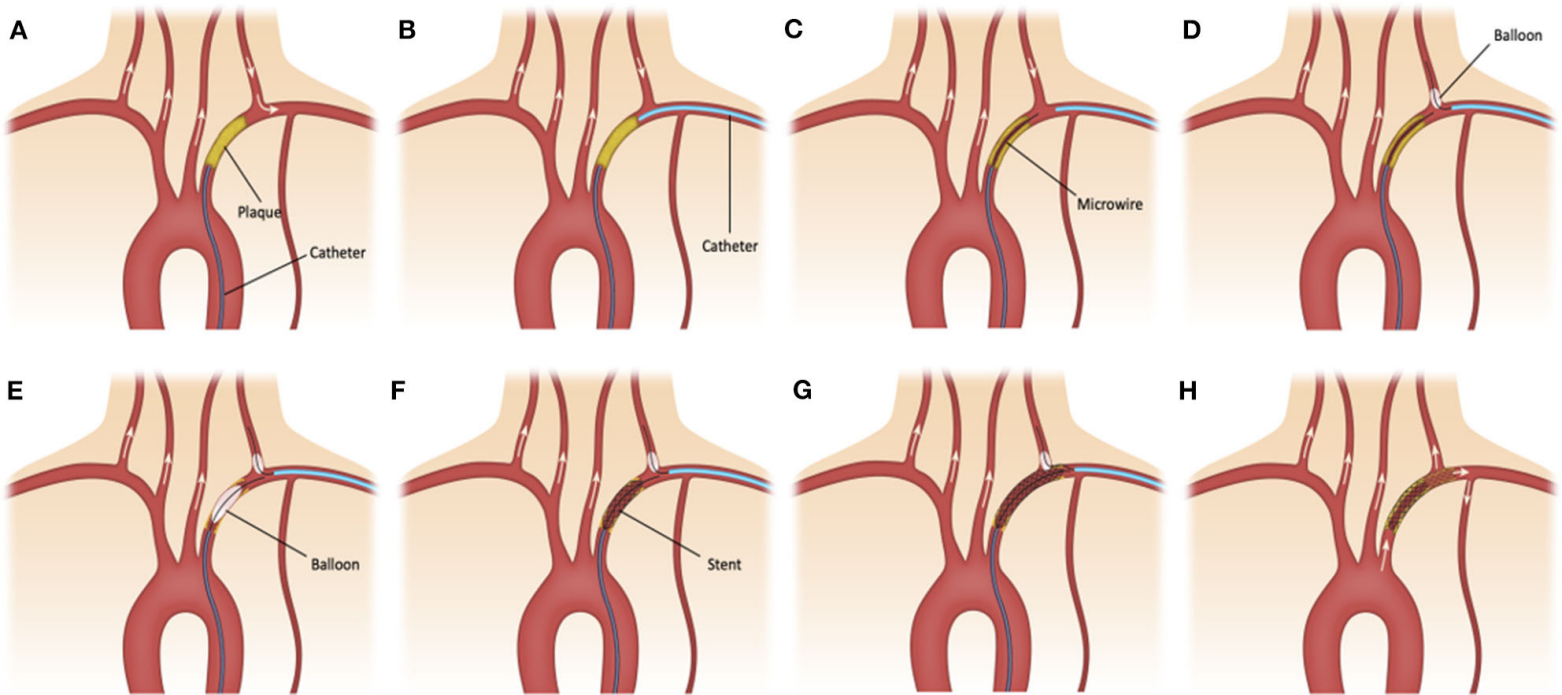
**(A)** 6F large bore sheath is advanced through the femoral access and placed in the proximal stump of the subclavian artery (SA). **(B)** 6F intermediate bore catheter is advanced through the radial access and placed distal to the SA lesion. **(C)** An anterograde channel is created across the SA lesion using a 0.014 in microwire. **(D)** The radial catheter is used to introduce a Scepter XC balloon catheter over a microwire in the ipsilateral vertebral artery (VA) for emboli protection. **(E–G)** Angioplasty and stenting of small and large atherosclerotic plaques is performed while inflating the balloon in the origin of the ipsilateral VA. **(H)** After successful revascularization of small and large (shadowed area) atherosclerotic plaques at the SA, the catheters and wires are removed.

The right common femoral access was obtained using 9 French short sheath which was connected to arterial line monitoring system. Radial artery ipsilateral to the SA lesion was accessed using ultrasound guidance and a 6 French slender short sheath was placed. All patients were adequately heparinized. A 6 French large bore guide catheter (Cook shuttle 087 [Cook Medical Inc, Bloomington, IN, USA] or Penumbra Neuron Max 088 [Penumbra Inc, Alameda, CA, USA) was advanced through the femoral access and placed in the proximal stump of the SA (Figure 1A). Six French intermediate bore catheter (Cook Envoy 070 or Penumbra Neuron 070) was advanced through the radial access and placed distal to the SA lesion (Figure 1B). Dual catheter contrast injection was performed to identify the length of the lesion. An anterograde channel is created across the SA lesion using a 0.014 microwire or 0.035 guide wire (Figure 1C). The radial catheter was used to introduce a Scepter XC balloon (Microvention Inc, Aliso Viejo, CA, USA) over a microwire to place it in the VA for protection from emboli during angioplasty and stenting of the SA (Figure 1D). The radial artery catheter was also used to create a retrograde channel across the SA lesion in cases when antegrade access could not be obtained. Once access was obtained to the SA lesion using a 0.014 wire, angioplasty followed by stenting was performed using 0.014 monorail balloons and stents while inflating the Scepter XC balloon in the origin of the ipsilateral VA. The position of the balloon was at the first straight segment of the VA to prevent any embolic debris to penetrate into the origin of the VA during the balloon inflation and the SAPTA deployment (Figures 1E–G). After successful revascularization of the SA, the catheters and wires were removed (Figure 1H). Femoral hemostasis was achieved with deployment of Terumo Angioseal (Terumo Medical Corp., Somerset, NJ, USA) closure device while radial hemostasis was achieved with application of Terumo TR band. Dual approach was preferred over single subclavian stenting based on proceduralist's preference and if concerns are present for increased risk of VA embolism.

### Assessment of Outcomes

Procedural information including type of arterial access, catheters, stents, and residual stenosis were collected from the review of the images and operative reports. Success of the procedure was defined as improvement in the caliber of the SA post intervention and improved antegrade flow on angiography with final residual stenosis of <20%. Periprocedural complications were defined as any stroke (hemorrhagic or ischemic), femoral or cervical or brachial or radial vascular injury, hematoma or infection at the site of entry, allergic reaction to contrast or kidney injury seen within 30 days of the intervention. At 3, 6, and 18 months follow-up, the clinical outcomes of interest were resolution of presenting symptoms and the modified Rankin Scale (mRS). CTA of the neck was done at 3 months follow-up to assess the patency of the SA. Follow-up complications were defined as any technical complication reported or seen at follow-up, such as in-stent restenosis, dissection, as well as secondary cerebrovascular accidents, TIA, and death.

## Results

A total of eight cases with SA stenosis/occlusion and SSS underwent SA stenting. The average age was 67.9 years (range 53–84 years). There were four males and four females. All patients had a history of hypertension and dyslipidemia. Four patients underwent the standard transfemoral anterograde approach, and the other four patients had the combined anterograde/retrograde approach. Procedure details and clinical outcome for all patients are summarized in Table 1.

**Table 1:** Endovascular hybrid anterograde and retrograde approach, materials, recanalization, and technical complications.

		**Stenosis grade and location**	**Catheters**		**Stent**	**Success^a^**	**Periprocedural complications^b^**	**Follow-up complications**
			**Femoral**	**Radial**				
Standard approach (femoral)	Patient 1	95% L SA	R groin: 6Fr Cook Shuttle	None	Stent BMS Carotid XACT 8–6 mm × 30 mm	Yes	None	None
	Patient 2	100% L SA	R groin: 6Fr Neuron Select BER catheter	None	Stent BMS VASC ZILVER 6Fr 80 cm/6 mm × 40 mm	Yes	None	None
	Patient 3	80% L SA	R groin: 6Fr 088 Neuron Max	None	9–7 × 30 mm Abbot Carotid Stent	Yes	None	SA stent occlusion 6-mo follow-up. Patient was retreated successfully. Patency at 3-mo.
	Patient 4	50% L SA	R groin: 6Fr Shuttle Select	None	Stent BMS Carotid XACT 8–6 mm × 30 mm	Yes	None	None
Combined approach (femoral/radial)	Patient 1	100%, L SA	R groin: 6Fr 088 Neuron Max	L radial: 6Fr Penumbra 070	9 × 7 × 30 Abbott carotid stent	Yes	None	None
	Patient 2	100%, L SA	R groin: 6Fr Cook Shuttle	L radial: 6Fr Envoy	VASC ZILVER STENT BMS 6 × 8 × 40	Yes	None	None
	Patient 3	95%, L SA	R groin: 6Fr Cook shuttle	L radial: 6Fr Envoy 070	VASC ZILVER Stent BMS 6 × 8 × 40	Yes	None	None
	Patient 4	100%, L SA	R groin: 6Fr 088 Neuro Max	L radial: 6Fr neuron 070	n/a	No	Contrast was observed at the subintimal level of the aortic arch.	Not available

*Legend: R, right; L, left; L SA, Left subclavian artery*.

a *Success defined as improvement in the caliber of the left subclavian artery post intervention with no residual stenosis and improved antegrade flow on angiography*.

b *Periprocedural complications defined as any kind of complication seen <30 days post intervention, such as hematomas, dissection*.

In the standard approach group, successful revascularization was achieved in all patients. One patient had a total occlusion, two patients had severe stenosis and one patient moderate stenosis. No periprocedural complications were observed. All stents were patent at 3-months CA. One patient developed an asymptomatic SA stent occlusion at 6-months follow-up. The patient was successfully retreated with SA SAPTA without any further complications.

**Figure 2:**
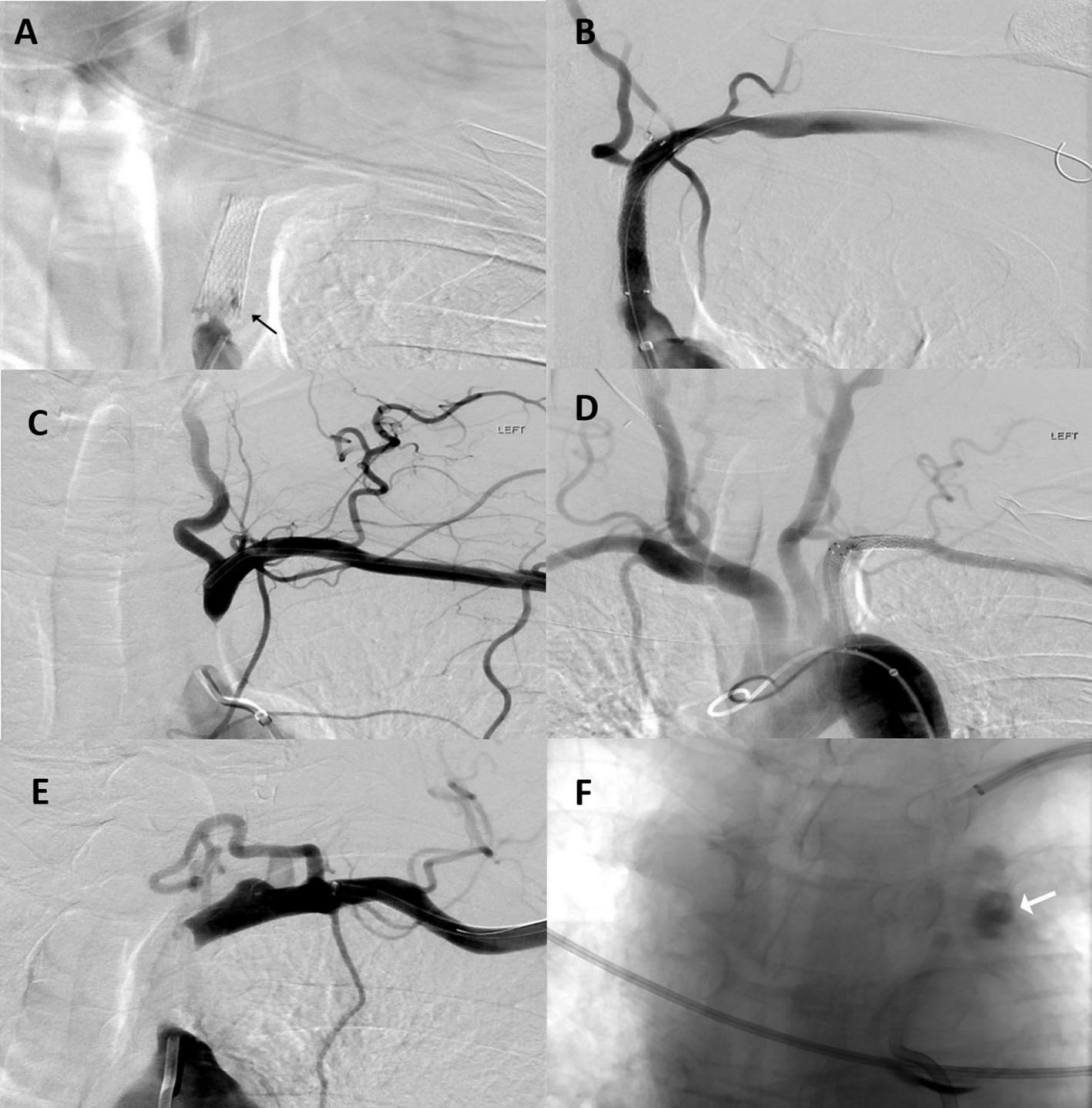
Patient 1: **(A)** Anteroposterior (AP) digital subtraction of angiography (DSA) with left proximal SA stent occlusion (black arrow). **(B)** Final angiographic results after stent assisted percutaneous transluminal angioplasty. Patient 2: **(C)** AP DSA with left proximal SA occlusion. **(D)** Final angiographic results after angioplasty and stent placement. Patient 4: **(E)** AP DSA with left proximal SA occlusion. Multiple attempts at recanalization were made but were unsuccessful. **(F)** During one these attempts; contrast extravasation was observed at the level of the aortic arch (white arrow).

**Figure 3:**
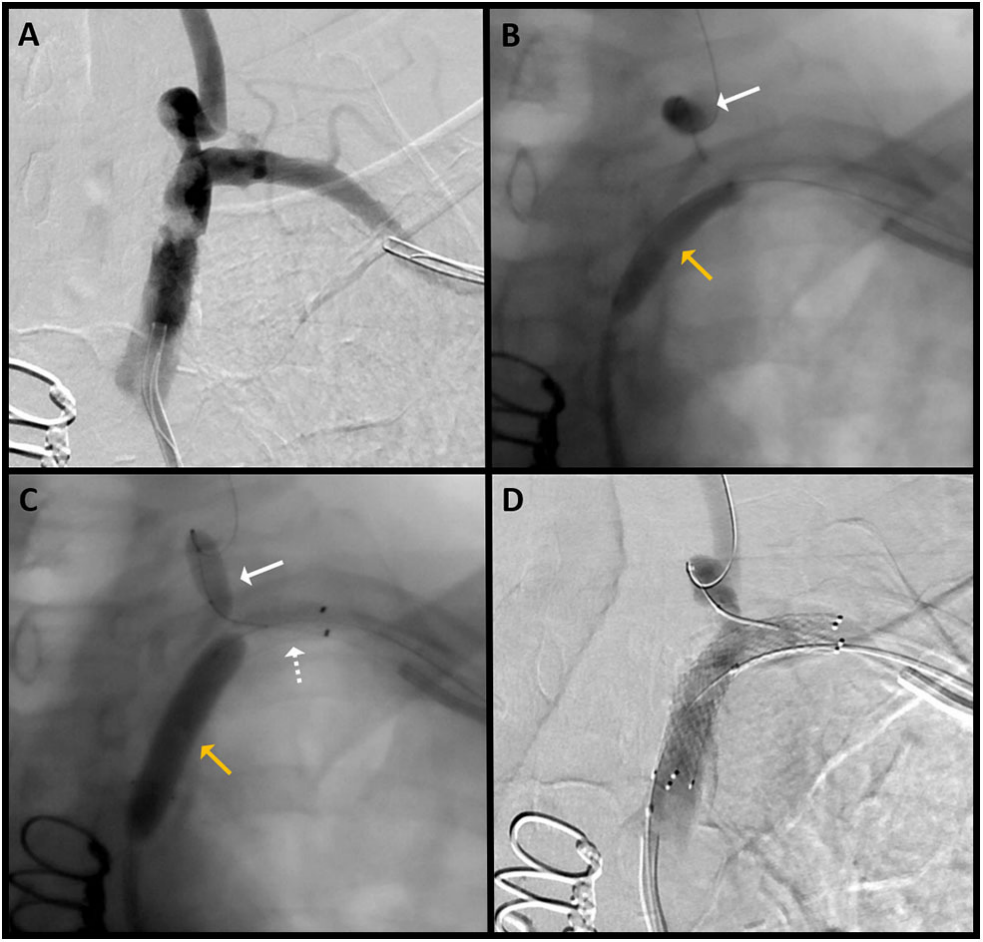
Patient 3: **(A)** AP DSA with severe stenosis of the left SA. **(B)** The radial catheter is used to introduce a balloon (white arrow) over a microwire in the left VA for emboli protection during pre-stent balloon angioplasty (yellow arrow). **(C)** Angiogram shows the stent (dotted white arrow) placed at the targeted lesion site; slow inflation of the SA balloon (yellow arrow) for post-stent angioplasty is performed, and the left VA balloon (white solid arrow) is again inflated for emboli protection. **(D)** Final angiographic results after angioplasty and stent placement.

In the dual approach group, successful revascularization was achieved in three out of the four patients. Pre- and post-revascularization DSA pictures can be visualized in Figures 2, 3. Three patients had a total occlusion of the SA and the fourth had a nearly occluded SA. It was difficult to create a channel in the unsuccessful case due to heavily calcified plaque burden. No perioperative ischemic events were identified. One subintimal dissection without clinical manifestation was reported as a periprocedural technical complication. For follow-up, only the information of the three successfully revascularized patients was available demonstrating resolution of SSS symptoms and no restenosis (Table 1).

The clinical history and outcomes of the combined approach patients are described in more detail below.

### Patient 1

Patient presented with 3 months history of intermittent dizziness and vertigo. DSA demonstrated left SSS with occlusion of left SA. Left SA revascularization using the above described combined approach was performed without complications. Dual antiplatelet therapy and high dose statin were recommended. Follow-up imaging after 3 months showed patency of the stent.

### Patient 2

Patient presented to the hospital with a 2-days history of left arm weakness and dysmetria, as well as some memory problems. DSA demonstrated total occlusion of the proximal left subclavian with evidence of subclavian steal phenomenon. Further history obtained was suggestive of clinical subclavian steal phenomenon, with left arm weakness on activity. Successful revascularization of the left SA with the above described technique was performed. There were no peri-procedural or follow up complications with resolution of symptoms at 3 months follow up. Dual antiplatelets and moderate intensity statin were continued for 3 months followed by mono-antiplatelet therapy life-long.

### Patient 3

Patient initially presented with scattered posterior circulation strokes. CTA neck showed left SA near-occlusion. Patient was complaining of intermittent dizziness and memory difficulties. DSA showed near occlusion of the SA with left SSS with retrograde flow in the ipsilateral VA. Left subclavian SAPTA as described above was performed with improved degree of stenosis. The procedure was without complication. Dual antiplatelet and high dose statin were continued. Three months follow up with CTA of the neck showed a patent SA.

### Patient 4

Patient presented with symptoms of left arm weakness. Workup demonstrated total occlusion of the left SA. Multiple attempts at recanalization using the above described technique were made but were unsuccessful. During one of these attempts, contrast extravasation was observed at the level of the proximal subclavian stump due to a dissection without catheterizing the true vessel lumen. By then, the amount of contrast as well as the amount of radiation exposure were maximized for the patient's age, so the decision was made to stop the procedure. Patient was advised to follow-up to discuss other treatment modalities but was lost to follow-up.

## Discussion

SA high-grade stenosis and occlusion can lead to a vascular phenomenon known as SSS, which can cause upper extremity claudication, posterior circulation transient ischemic attack, stroke, syncope, and vertigo. Available treatment options include percutaneous transluminal angioplasty (PTA), direct surgical recanalization, or surgical bypass. Current guidelines recommend endovascular approach as the first line in patients with atherosclerotic lesion of the upper extremities (17). This is based on multiple studies that have shown the technical success rate of PTA to be high and similar to that of surgical treatment but also less invasive with a low rate of major complications (3–9, 18). PTA for SA stenosis is performed widely as an alternative to surgery, and the technique was first described in the early 1980's in several small series of patients (19, 20).

The success rate for PTA is lower for total occlusion than for stenosis, especially in patients with SSS (21, 22). Liu et al. (7) reported that the technical success of PTA for SA total occlusion was achieved in 77.6% patients, with complications occurring in 6% of them. Babic et al. studied 56 patients with total occlusion of SA (6) and showed that the primary patency rates after 1 and 3 years were 97.9 and 82.7%, respectively. Some researchers propose that endovascular technology is preferable in patients with high-grade stenosis while open bypass is preferable for SA total occlusion (9); others showed that endovascular therapy is efficient for both with no difference between patients who had stenosis and those who had occlusions based on several case series (6, 8, 21).

The most concerning complication of endovascular treatment of SSS is distal VA embolism. Literature shows a 1–5% risk of stroke during SAPTA of SA (14–16). This incidence rate of posterior circulation vascular events coincides with the 5% stroke rate in the 1st year observed after endovascular therapy of a VA stenosis (23).

The presence of distal embolic debris has been reported in a series of 14 patients undergoing VA stenting using distal protective filters. Filters captured debris in all patients, and this occupied between 0.1 and 22% of the filter area (24). Furthermore, it has been observed an 8.3% presence of emboli signals in the VA under Doppler ultrasound continuous insonation of patients treated with SA PTA (25). Unfortunately, it is not described whether these patients suffered from SSS manifestations despite they all depicted high grade of stenosis/occlusion.

SSS is generally considered as a protective factor for distal emboli due to the presence of retrograde flow in the ipsilateral VA. However, it is important to consider the intermittency at which SSS can present, as the majority of SSS are asymptomatic with precipitation of retrograde flow upon exertion of the ipsilateral upper extremity (2). Thus, this flow reversal would not be protective if there is no steal at rest (26). Nevertheless, it has been observed the presence of posterior circulation strokes during subclavian PTA in the presence of SSS in a range of 1.0–1.7% cases without the use of distal protective devices (8, 27). This leads to the idea that the supposed retrograde flow protection could be overestimated. In our series, with the presence of intermittent VB symptoms and documented SSS, we opted to decrease the risk of VA embolism using distal protection.

Unlike carotid artery angioplasty and stenting during which protection against distal embolization is obtained by either deployment of distal filter device or proximal flow arrest, SA angioplasty and stenting traditionally does not allow for either of these techniques due to the need of covering the VA to obtain an adequate distal coverage of the plaque.

One of the protective techniques against distal embolisms described by Yamamoto et al. is by using the non-compliant Optimo balloon catheter (Tokai Medical Products, Aichi, Japan) through the ipsilateral radial artery (18, 28). In their case series, Yamamoto et al. (18) placed the 6-French Optimo balloon catheter in the SA proximal to the origin of the VA after introducing it through an ipsilateral transradial access. They inflated the balloon around the catheter while performing antegrade angioplasty and stenting of the affected SA for distal emboli protection. Nakamura et al. (28) in their paper described a similar technique. They described two cases in which brachial artery access was established for the insertion of a balloon-tipped occlusion catheter (Optimo occlusion balloon catheter) into the SA proximal to the VA. Even though this technique appears to provide protection against distal emboli protection, there is concern that during inflation of this non-compliant balloon at the tip of the catheter, it performs angioplasty of that segment of the proximal VA and could be its own source of emboli. Secondly, and more importantly, in cases of long lesions of the SA (covering the origin of the left VA), there is not much room to perform the planned SAPTA and adequately cover the plaque distally with a stent due to the large size of the balloon. Lastly, the Optimo catheter is not found in the US market.

The use of distal filters in the VA during subclavian/vertebral stenting has been previously described with technical success and without complications (24, 29–31). The advantage of distal filters over balloons relies on the preservation of the antegrade cerebral flow throughout the procedure. However, the larger and more rigid delivery systems difficult their navigation through tortuous vessels with the risk of vessel spasm and/or dissection (32). Additionally, there is the risk of embolization of particles smaller than the size of the pores (33).

Herein, we used the transradial catheter to introduce a Scepter XC balloon (Scepter XC™, Microvention, Inc., Tustin, CA, USA) over a microwire to place it in the ipsilateral VA for protection from emboli during angioplasty and stenting through a femoral route. The placement of this compliant balloon in the ipsilateral VA provides adequate protection from distal emboli without itself performing any meaningful angioplasty and producing new source of emboli. Since it is a 0.017-inch catheter, it leaves ample room to perform angioplasty and stenting of long lesions of the SA (covering the origin of the left VA), with easy withdrawal of the catheter once deflated after being jailed in the subclavian stent. A similar technique was previously described by Sadato et al. (34) where they used a different balloon catheter for distal protection (Multilumen balloon catheter; Clinical Supply Co., Hajima, Gifu), which is not found in the US market.

Our case series along with those of Yamamoto, Nakamura, and Sadato (18, 28, 34) are the few to describe a combined antegrade and retrograde endovascular approach to SA SAPTA. Our paper will serve as a reminder to young endovascular surgeons of the effectiveness, safety, and feasibility of this technique for SA recanalization now using new compliant small dual lumen balloons. One of the advantages of this technique is the ability to provide protection from distal emboli even in the subclavian plaque extends beyond the vertebral ostium as discussed above. The second advantage is it allows for double simultaneous injection through the transfemoral and transradial catheters to identify the length of the lesion and direction of the proposed wire route in cases of occlusion of the SA. Most importantly, in cases of occluded subclavian arteries in which it is not possible to cross the occluded lesion in antegrade fashion, it provides for potential retrograde access to the lesion.

There are limitations in our study. The retrospective nature of the study can lead to biases. The small sample size is not large enough to demonstrate any beneficial effect on embolic complications. Only four of our eight patients with SSS underwent the dual approach technique. The technique was used in patients with higher degree of stenosis and occlusions when the neurointerventionalist thought that there was an increased risk of vertebral emboli due to plaque proximity to the vertebral ostium. The use of the dual approach can lead to increase potential for complications including radial artery injury, temporary and permanent occlusion, and compartment syndrome.

## Conclusion

We conclude that a combined retrograde/antegrade endovascular approach along with use of balloon catheters as protective devices against distal embolism in the ipsilateral VA should be considered during SA recanalization in the setting of SSS. This technique could be an option in complete occlusions prior to more aggressive alternatives such us bypass. Further study of this technique in larger prospective multicenter registries is warranted before widespread adoption to better evaluate its potential protective effect on distal embolization during SA stenting.

## Data Availability Statement

The original contributions presented in the study are included in the article/supplementary material, further inquiries can be directed to the corresponding author/s.

## Ethics Statement

The studies involving human participants were reviewed and approved by University of Iowa Institutional Review Board. Written informed consent for participation was not required for this study in accordance with the national legislation and the institutional requirements. Written informed consent was obtained from the individuals/next of kin for the publication of any potentially identifiable images or data included in this article.

## Author Contributions

RF, SD, AAM, and SO-G designed and defined the intellectual content of the study. SD and SO-G performed the clinical studies. AM-R, MF, CZ, DQ, and AAM contributed with data acquisition. RF, SD, and SO-G wrote and prepared the manuscript. DH, JR, ES, CD, and SO-G provided critical review of the study. All authors reviewed and approved the final version.

## Conflict of Interest

The authors declare that the research was conducted in the absence of any commercial or financial relationships that could be construed as a potential conflict of interest.

## Abbreviations

SSS: Subclavian steal syndrome
SA: subclavian artery
SAPTA: stent assisted percutaneous transluminal angioplasty
DSA: digital subtraction of angiography
PTA: percutaneous transluminal angioplasty.
